# Occipitocervical fusion complicated with cerebellar abscess: a case report

**DOI:** 10.1186/s12891-020-3157-0

**Published:** 2020-02-28

**Authors:** Cheng-Chi Lee, Yu-Tse Liu

**Affiliations:** 1 Department of Neurosurgery, Chang Gung University, Linkou, 5 Fu-Shin Street, Gui-Shan Dist, Taoyuan City, Taiwan, Republic of China; 2 Chang Gung Memorial Hospital, Linkou, Chang Gung University, Taoyuan, Taiwan, Republic of China

**Keywords:** Occipitocervical fusion, Cerebellar abscess, Complications, C1 laminectomy, Atlantoaxial subluxation

## Abstract

**Background:**

Occipitocervical (OC) fusion is indicated for OC instability and other conditions. Surgical complications include infection, malunion, and instrument failure.

**Case presentation:**

We described a patient who underwent OC fusion and subsequently developed complication of cerebellar abscess and obstructive hydrocephalus. A 63-year-old male patient had been suffering from long-term neck pain and limb numbness and weakness. Cervical spine examination revealed tight stenosis at C1 level and instability in the C1-C2 joints. A C1 laminectomy with OC fusion was performed, and the patient was discharged. Unfortunately, a few days later, he went to the emergency department and complained of persistent dizziness, vomiting, and unsteady gait. Computed tomography (CT) and magnetic resonance imaging (MRI) images revealed a suspicious cerebellar abscess formation and hydrocephalus. Furthermore, CT images indicated that the left screw was loose, and the diameter of the right screw hole was much larger than the size of the screw. Besides, inappropriate length of the screw penetrated the occipital bone and may cause the disruption of dura mater. The patient underwent external ventricular drainage first, followed by abscess drainage and C1-C2 fixation a few days later. He was discharged without any further neurological deficits or infectious problems. The patient recovered with intact consciousness, full muscle strength, and improved numbness throughout the extremities, with a Nurick grade of 1. A follow-up magnetic resonance imaging at 3 months after surgery revealed near total resolution of the abscess. Inform consent was obtained from this patient.

**Conclusions:**

Carefully conducting the procedure using the most tailored approach is essential to successful surgery, but this rare complication should always be kept in mind.

## Background

For C1-C2 instability, C1-C2 fixation is a common and well-developed technique. However, this procedure is more challenging and has higher incidence of the risk of damaging vertebral artery (VA) due to the complexity of C1-C2 [1–3]. Instead, some surgeons will choose occipito-cervical (OC) fixation and skip C1-C2 instead of C1-C2 instrumentation to avoid the high risk of injury to the VA. There are some postoperative disadvantages of OC fixation, such as reduced cervical mobility, difficulty in swallowing, postoperative neck stiffness, etc. [4–8]. Possible complications of OC fixation includes VA injury, screw loosening, neurological deterioration, bone fusion failure, cerebellar infraction, nerve or cord injury, and wound infection [9–11]. To the best of our knowledge, OC fusion complicated by cerebellar abscess formation has not been reported thus far. Thus, we reported a patient who underwent OC fusion for C1-C2 subluxation, and subsequently resulted in cerebellar abscess and hydrocephalus complications.

## Case presentation

**Fig. 1:**
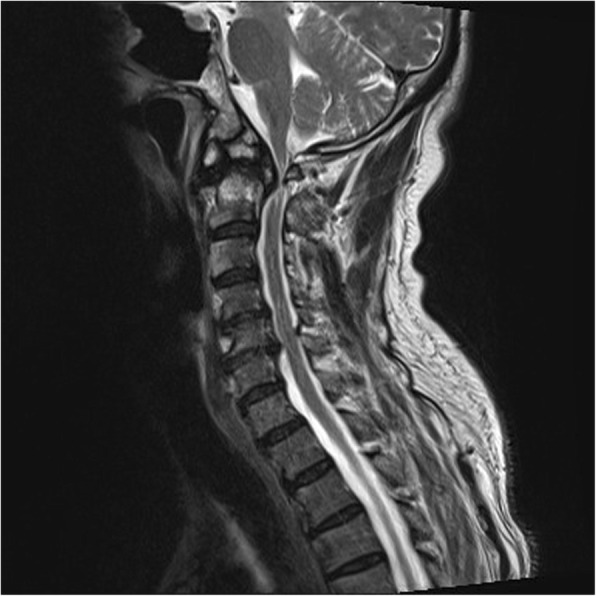
MRI image of hypertrophic ossification at the odontoid process, tight stenosis at the C1, and edematous changes at the spinal cord

**Fig. 2:**
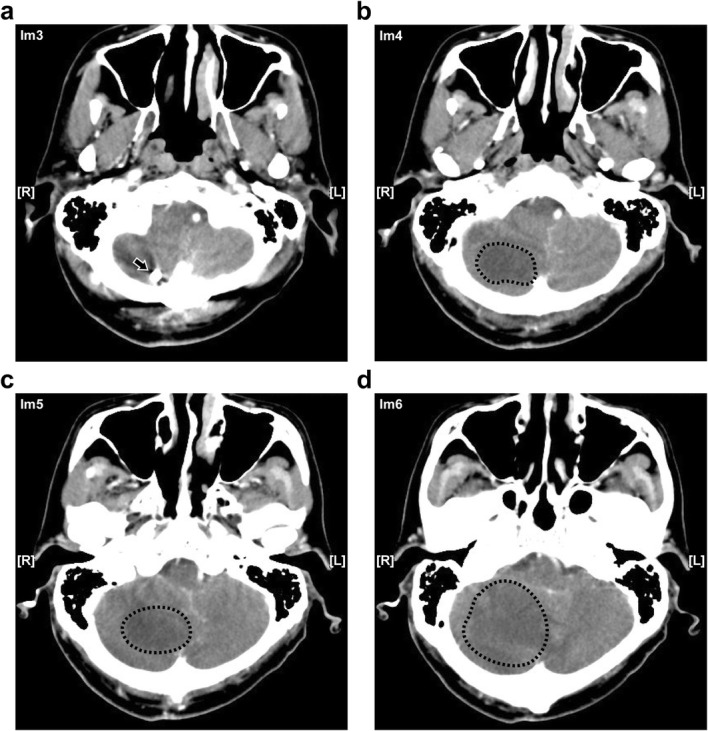
The axial images of four continuous CT of the patient with cerebellar abscess. The four continuous CT images from a to d represented continuous section from the base of the skull to top. The arrow in (**a**) indicated the position where the screw penetrates the dura mater. Circled area was indicated as brain abscess

**Fig. 3:**
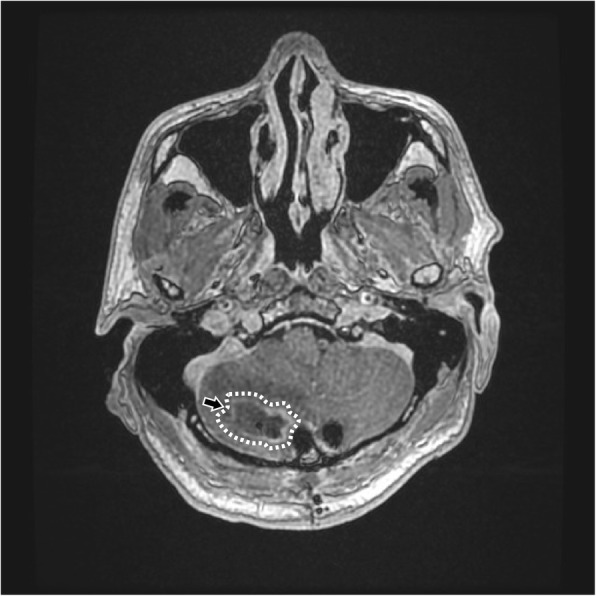
The appearance of cerebellar abscess at MRI image. MRI confirmed the diagnosis of a 2.5 cm of a faint ring-enhanced cerebellar abscess based on T1-weighted images with contrast medium. Circled area was represented as brain abscesses

**Fig. 4:**
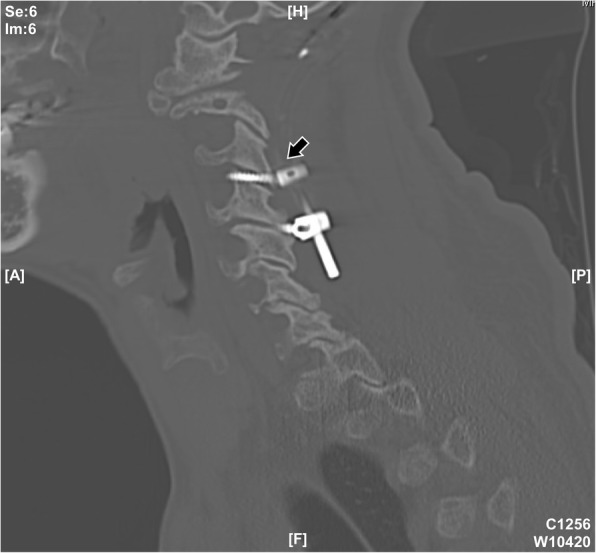
Possible VA injury by C3 screw. The arrow indicated that the C3 screw has been inserted into the transverse foramen, which may cause VA injury

**Fig. 5:**
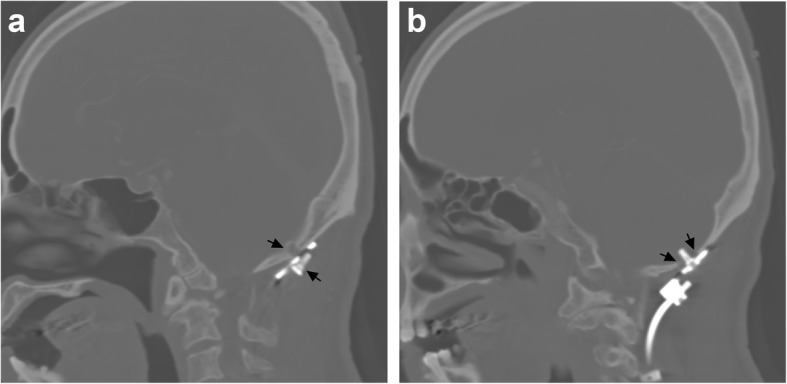
CT sagittal images of the failure of the OC fusion instruments. (**a**) The arrow showed the dislodgement of the occipital screw and the screw hole. The screw was loose and not in their original position. (**b**) The arrow indicated the oversized diameter of the screw hole. The diameter of the screw hole is much larger than the size of the screw, indicating that the screw has no fixing effect

**Fig. 6:**
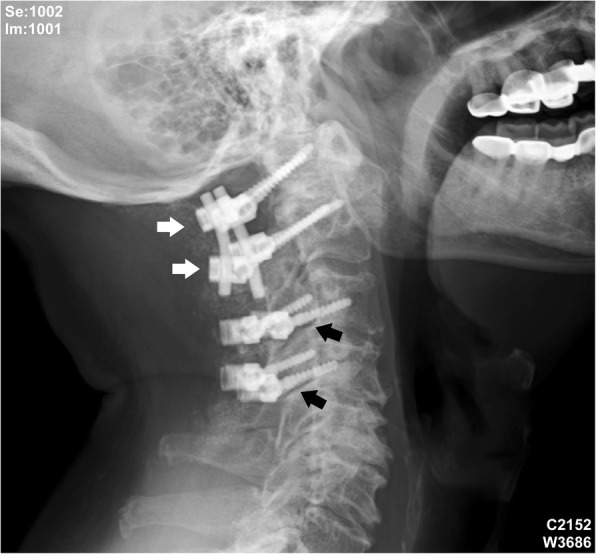
CT image after C1-C2 instrumentation. The white arrows indicated the newly operated C1-C2 instruments, and the black arrow indicated the original occipito-C3-C4 fixation instruments

**Fig. 7:**
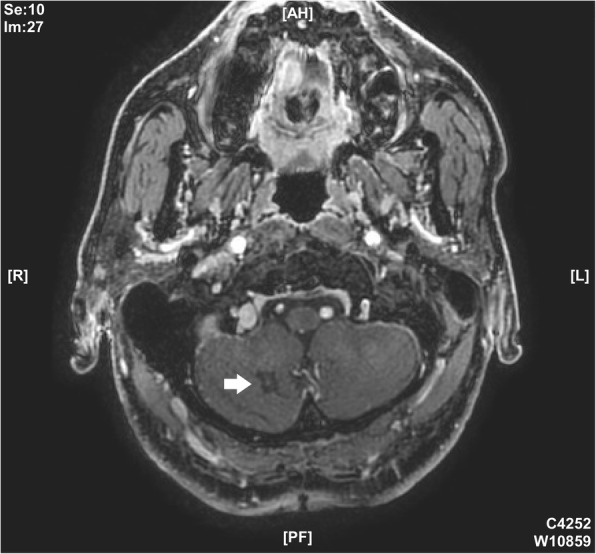
MRI image of cerebellar abscess resolution after surgery.MRI image showed that abscess in the right cerebellum was almost completely resolved 3 months after surgery. The arrow indicated the abscesses that has not resolved

A 63-year-old male patient had been suffering from long-term neck pain and limb numbness. The dynamic flexion-extension view revealed the C1-C2 instability, and magnetic resonance imaging (MRI) of the cervical spine revealed hypertrophic ossification at the odontoid process, resulting in tight stenosis at C1 level and spinal cord edema (Fig. 1). C1 laminectomy and OC fixation using the screw-rod system were performed by an orthopedic surgeon in another hospital. To avoid the risk of VA injury, the surgeon chose occipito-C3-C4 fixation instead of C1-C2 fixation. The patient was discharged after 1 week, but a few days later, he began to experience persistent intolerable dizziness and vomiting. He went to the emergency department of our hospital. Physical examination revealed that his consciousness was clear and alert and had no change in muscle power compared to the status before surgery. He had no fever. However, the blood test showed a slight increase in leukocytosis (11,800 per mm^3^) and C-reactive protein level (< 5 mg/dl), indicating that the infection was in the early stage. Furthermore, the patient has symptoms of unsteady gait and right-sided dysmetria, suggesting that the patient may have a cerebellar lesion. Computed tomography (CT) further revealed a significant signal enhancement around the occipital screw (arrow in Fig. 2a) and a hypodense lesion in right cerebellar hemisphere (circled area in Fig. 2b-d). Although the presence of the screw interfered with CT image, it is still found that the position of the screw was the origin of brain abscess. In addition, the screw of inappropriate length were observed to penetrate through the occipital bone (Supplementary Figure 1). Since the CT image showed only a mild patchy enhancement, the lesion is more likely to be a cerebritis rather than a neoplasm. Therefore, we speculated that it should be a central nervous system (CNS) infection, and the patient was initially prescribed broad-spectrum antibiotics, including Vancomycin and Ceftazidime. Although the local metallic implants interfered with the interpretation of MRI, subsequent MRI confirmed the lesion as a 2.5 cm faint ring-enhanced cerebellar abscess based on the T1-weighted images with contrast medium and on the diffusion weighted imaging sequences (Fig. 3). A few days later, the patient became drowsier, so we decided to perform emergent external ventricular drainage (EVD) first to divert CSF and release intracranial pressure. When the wall of abscess is firmly formed, a second stage of suboccipital craniectomy will be performed to drain the brain abscess, and the implants will also be readjusted at the same time. One week after EVD surgery, the patient underwent an elective operation to drain the cerebellar abscess, debride the necrotic tissue, and inspect and readjust the hardware. Preoperative CT angiography (CTA) was conducted to evaluate the relationship between the VA and the planned trajectory, and it excluded a high-riding VA, demonstrating a safe trajectory for bilateral C2 transpedicular screws. In addition, the CTA scan also revealed that additional surgical changes in the positions of the left C3 and C4 screws may not be appropriate as it may breach the transverse foramen and damage the VA (Fig. 4). Moreover, the CT sagittal images further showed dislodgement of the occipital screw (Fig. 5a) and oversized diameter of the screw hole (Fig. 5b), indicating the failure of the instruments. Considering that the infectious status was limited to only around the occipital and cerebellar area and the persistent C1-C2 instability, therefore, in addition to the debridement and drainage of the abscess, the next step is to remove the previous hardware and reintroduce the C1 lateral mass and C2 transpedicular screws. In order to avoid the risk of VA injury when removing C3 and C4 screws, we left these screws in position. Next, we enlarged the hole of the right occipital screw and confirmed the abscess by echography. No purulent discharge was found, but necrotic tissue was found and debrided. The C1 lateral mass and C2 transpedicular screws were safely introduced under fluoroscopic guidance without VA damage (Fig. 6). The postoperative course was uneventful, and the symptoms of dizziness, unsteady gait, and dysmetria improved gradually. A few days later, the EVD was removed. No pathogens were detected from the final tissue and CSF cultures, antibiotics were prescribed for the patient for 8 weeks, and the patient was discharged uneventfully. An MRI examination at 3 months postoperatively revealed that the cerebellar abscess almost completely subsided (Fig. 7).

## Discussion and conclusion

OC fusion is indicated for OC instability and some cases of C1-C2 subluxation, and it promises a satisfactory fusion rate and neurologic improvement [9]. The related complications include pneumonia, deep venous thrombosis of the lower extremity, wound infection, CSF leakage (dural injury), malunion, instrument failure, and VA injury [9, 12–14]. In the literature review, we found a report of intracranial infections and brain abscesses in patient using the Halo jacket for cervical spine fixation [15]. However, to date, no cerebellar abscess has been reported due to OC fixation. In our patient with true C1-C2 instability, the orthopedic surgeon did not first arrange a CTA study to evaluate the feasibility of C1-C2 instrumentation. Instead, he chose a simpler OC fusion procedure that eventually resulted in rare serious complications of cerebellar abscess and caused obstructive hydrocephalus, screw loosening, and inappropriate trajectory and position on the left-sided C3 and C4 screws. Furthermore, the postoperative plain film revealed that the occipital plate did not fit the curve of the occiput, which may have been the cause of the screw loosening (Supplementary Figure 2). Moreover, the length of the original right occipital screw seemed to be too long and penetrated the dura mater of the posterior fossa (Fig. 5b and Supplementary Figure 1), resulted in an increased risk of intracranial infection and eventually abscess formation.

OC fusion provides satisfactory arthrodesis and biomechanical stability to treat degenerative diseases, trauma, tumors, and inflammatory processes. The screw-rod system has more favorable outcomes and less adverse complications than other fusion techniques [16]. However, no matter which system is used, it is obvious that OC fusion techniques are much more easily and quickly performed than C1-C2 fusion when considering the risk of VA injury. Nevertheless, compared to OC fusion, C1-C2 instrumentation is the most straightforward procedure for C1-C2 subluxation and provides the most powerful rigid fixation without sacrificing too much range of motion. However, no matter which fusion technique is performed clinically, familiarization with every step of instrumentation is the most critical part of dealing with high cervical surgical procedures. This also includes determining the optimal and safe trajectory by intraoperative fluoroscope, choosing the appropriate length and type of screws, and avoiding infection at the surgical site. Furthermore, in addition to reduce surgery-related complications, we recommend the most appropriate surgical approach should be adopted based on the comprehensive acquisition and evaluation of the patient’s preoperative information.

In conclusion, OC fusion complicated with cerebellar abscess and resultant obstructive hydrocephalus has never been reported. Choosing the most tailored approach and being familiar with each surgical step are essential to avoid major complications.

## Supplementary information

**Additional file 1: Figure S1.** Arrow indicates image evidence of improper length of screws penetrating occipital bone after initial OC fusion.

**Additional file 2: Figure S2.** A postoperative plain film of the initial OC fusion indicated that the occipital plate did not conform to the occipital curve.

## Data Availability

All data generated or analysed during this study are included in this published article and its supplementary information files.

## Abbreviations

CNS: Central nervous system
CSF: Cerebrospinal fluid
CT: Computed tomography
CTA: CT angiography
EVD: External ventricular drainage
MRI: Magnetic resonance imaging
OC: Occipitocervical
VA: Vertebral artery
