# Supplementary figures and images for: Occipitocervical fusion complicated with cerebellar abscess: a case report
Source: BMC Musculoskelet Disord. 2020 Feb 28;21:129. doi: 10.1186/s12891-020-3157-0 (PMC7049210; doi:10.1186/s12891-020-3157-0)

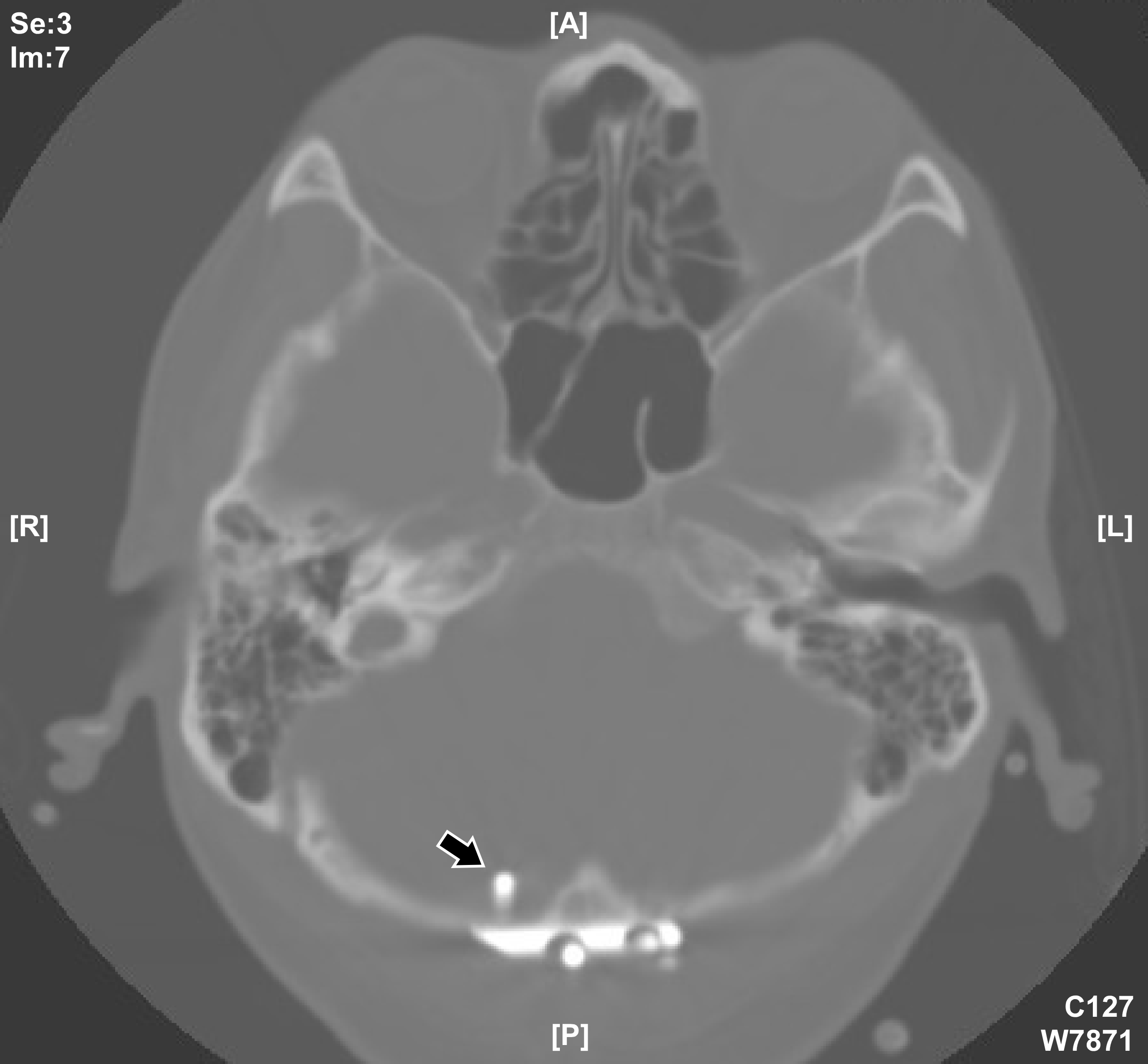

Supplement: Supplementary file 1 — Additional file 1: Figure S1. Arrow indicates image evidence of improper length of screws penetrating occipital bone after initial OC fusion. [file 12891_2020_3157_MOESM1_ESM.jpg]

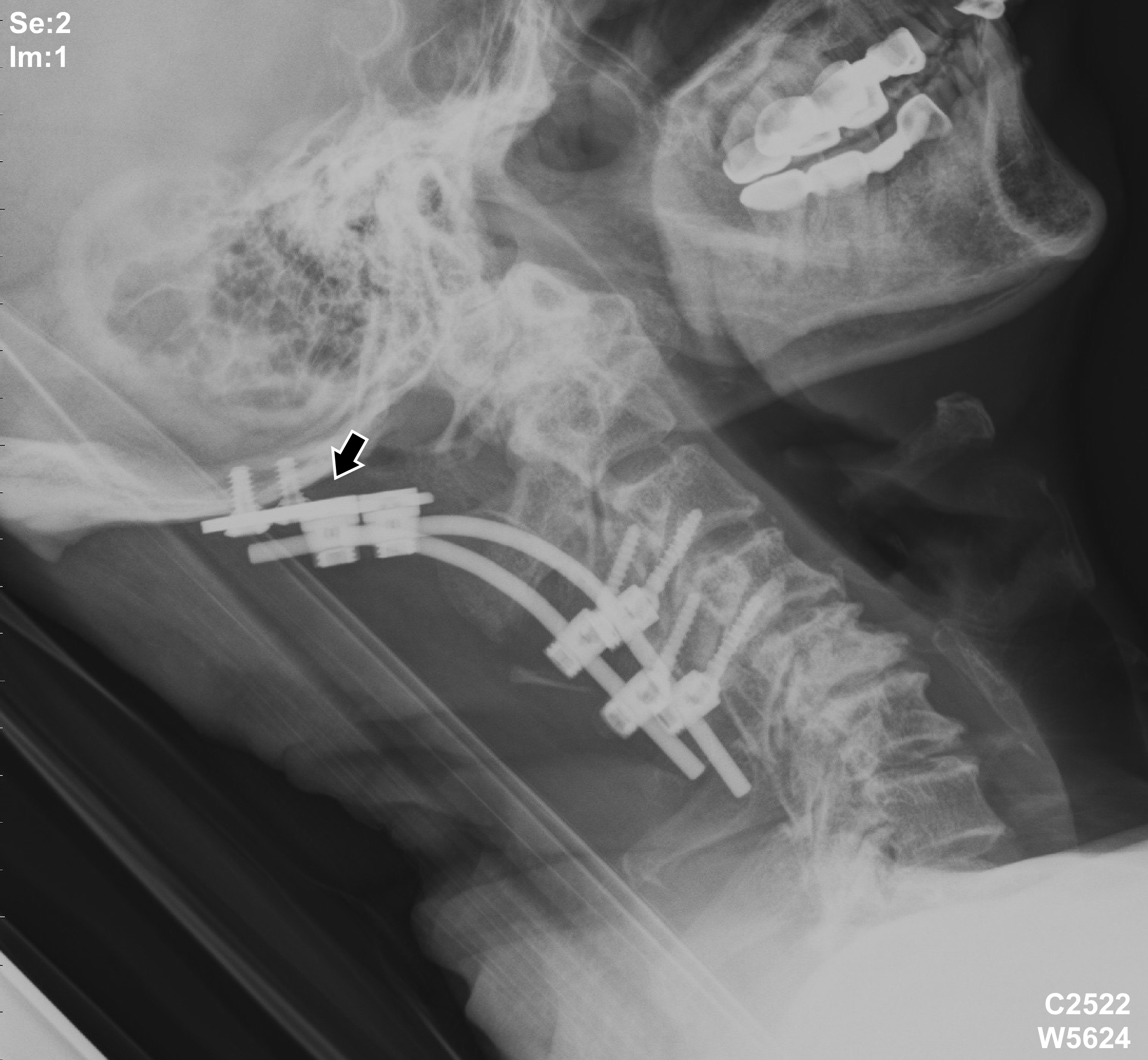

Supplement: Supplementary file 2 — Additional file 2: Figure S2. A postoperative plain film of the initial OC fusion indicated that the occipital plate did not conform to the occipital curve. [file 12891_2020_3157_MOESM2_ESM.jpg]
